# Gone with the Wind: Conceiving of Moral Responsibility in the Case of GMO Contamination

**DOI:** 10.1007/s11948-015-9744-z

**Published:** 2016-01-06

**Authors:** Zoë Robaey

**Affiliations:** Department of Values, Technology and Innovation, Faculty of Technology, Policy and Management, Delft University of Technology, Jaffalaan 5, 2628 BX Delft, The Netherlands

**Keywords:** Genetically modified organisms, Contamination, Moral responsibility, Social experimentation, Epistemic virtues

## Abstract

Genetically modified organisms are a technology now used with increasing frequency in agriculture. Genetically modified seeds have the special characteristic of being living artefacts that can reproduce and spread; thus it is difficult to control where they end up. In addition, genetically modified seeds may also bring about uncertainties for environmental and human health. Where they will go and what effect they will have is therefore very hard to predict: this creates a puzzle for regulators. In this paper, I use the problem of contamination to complicate my ascription of forward-looking moral responsibility to owners of genetically modified organisms. Indeed, how can owners act responsibly if they cannot know that contamination has occurred? Also, because contamination creates new and unintended ownership, it challenges the ascription of forward-looking moral responsibility based on ownership. From a broader perspective, the question this paper aims to answer is as follows: how can we ascribe forward-looking moral responsibility when the effects of the technologies in question are difficult to know or unknown? To solve this problem, I look at the epistemic conditions for moral responsibility and connect them to the normative notion of the social experiment. Indeed, examining conditions for morally responsible experimentation helps to define a range of actions and to establish the related epistemic virtues that owners should develop in order to act responsibly where genetically modified organisms are concerned.

## Introduction

In ethics of technology and ethics of engineering, many scholars have asked how and why to ascribe moral responsibility to agents in forward- and backward- looking ways (see Vincent et al. 2011). However, new technologies bring new challenges to the field of engineering ethics. In this paper, I consider the case of genetically modified organism (GMO) contamination, which challenges the ascription of moral responsibility to agents based on the condition of knowledge. Indeed, how can we speak of the ascription of moral responsibility regarding a technology when its effects are very difficult to know or simply unknown? In this paper, I hope to contribute to the fields of engineering ethics and responsibility theory by exploring this question through the problem of GMO contamination.

There has been much discussion about the contamination that results from the use of GMOs in agriculture, which may create different types of harm. Firstly, there is economic harm to farmers who might lose organic certification because of the presence of GMOs in their fields; or, conversely, seed developers with certain rights over these seeds might lose out on expected profits when ‘escaped’ or ‘duplicated’ seeds are used without their permission. Many legal costs are involved in sorting these issues out. Secondly, there is environmental harm in the sense that agricultural biodiversity (and even biodiversity more generally) may be affected by the presence of new seeds or horizontal gene transfer in ways that are still unknown. Lastly, just as the effects of GMO contamination on the environment are currently unknown, unwanted and unknown GMOs in food may impact human health. This is highly speculative, as there is no uncontested evidence that points to such effects. However, past experiences with new technologies have shown that they should not simply be, so to speak, innocent until proven guilty and that we should show some precaution and humility when dealing with these new technologies (Stirling 2007). ISAAA (2015) reports that, “in 2014, a record 181.5 million hectares of biotech crops were grown globally—[…] more than 100-fold gain since 1.7 million hectares were planted in 1996.” Their increasing popularity make the question of responsibility ascription for technologies with unknown effects ever more important.

In answering this question, I first introduce some state of the art considerations with regard to knowledge and responsibility theory. I then present my proposal to deal with the hazards of GMOs by ascribing forward-looking moral responsibility to owners, and I explain how this proposal runs into problems when it comes to contamination. By drawing from experiences in the field of nuclear energy, as well as from the normative notion of social experimentation, I suggest practical solutions. I then return to the original problem of knowledge and responsibility to finalize my proposal about how owners are morally responsible for new technologies when their effects are unknown.

## Knowledge, Ignorance and Responsibility

Scholars distinguish between forward- and backward-looking moral responsibility: the latter as a way to establish moral responsibility after the fact and the former as ‘prospective’ way. Backward-looking moral responsibility can usually be ascribed to an agent if the following conditions are fulfilled, at least to some extent: freedom of action, foreseeability, wrongdoing, causality, and capacity (Doorn 2012; Nihlén Fahlquist et al. 2014). Freedom of action and capacity mean that an agent was not coerced into performing an action and that this agent has the means to perform that action. The three remaining conditions, foreseeability, wrongdoing, and causality, are linked to having *knowledge* about an action *α* and its results. Foreseeability implies the ability to anticipate things that will happen as a result of an action *α*. Wrongdoing also implies that there is knowledge that an action *α* might go against some norms. Causality means that after the fact, we are able to trace the causes of an action *α* and link them to the consequences of that action.

If an agent possesses only limited knowledge about an action *y*, we encounter the problem that foreseeability, wrong-doing, and causality become conditions that are difficult to judge when we assess her backward-looking moral responsibility, e.g. a farmer planting GMOs that are later found to be harmful. It would seem wrong to argue that if there are indeed limits to these conditions, then there is no moral responsibility on the part of the agent who has taken the action *y*.

These considerations are for backward-looking responsibility and knowledge, so what about the forward-looking variety? Van de Poel (2011b) argues that for at least two types of backward-looking moral responsibility, blameworthiness and accountability, there needs to be forward-looking moral responsibility ascription in the first place because moral responsibility is a relational concept. This does not include duties, as duties are specific actions for a specific individual and responsibilities are only concerned with a desired state of affairs, regardless of the individual who achieves it. The relational aspect of forward- and backward-looking moral responsibilities means that you cannot deem an agent blameworthy or accountable for an action that she did not have a forward-looking moral responsibility to perform before something went wrong and made her blameworthy or accountable. Therefore, a lack of (or limits in) knowledge will have an impact on the conditions for backward-looking moral responsibility, but it does not remove moral responsibility from an agent altogether.

This is quite a strong statement, because it does not remove responsibility from unknowing agents. Indeed, not knowing does not free someone from moral responsibility if she had a forward-looking moral responsibility in the first place. In terms of forward-looking responsibility, two types are identified in the literature: responsibility-as-virtue and responsibility-as-obligation (see Vincent et al. 2011). Although these distinctions can be challenged, this is not the purpose of my paper. Rather, I mention them here because the remainder of this paper will focus on responsibility-as-virtue. Van de Poel concludes his chapter on the link between forward- and backward-looking moral responsibility with a thought I would like to expand on. He writes, “Another direction in which the account can be extended is […] by incorporating the role of responsibility-as-virtue” (2011b, p. 51). Responsibility-as-virtue is defined as an agent having “the disposition (character trait) to act responsibly” (Van de Poel 2011b, p. 39). But what does having the disposition to act responsibly mean? Also, is there only one character trait that renders an agent morally responsible? This definition is very broad and therefore opens the door for exploring the concept of responsibility-as-virtue in the context of engineering ethics.

Being morally responsible with a lack of complete knowledge is another way of talking about culpable ignorance. Fitzpatrick’s work (2008) is prominent in the ethical debate over whether there is culpable ignorance outside akrasia. The proponents of the akratic condition argue that there is only culpable ignorance if it can be traced back to an akratic action, i.e. if an agent, at some point, exhibited weakness of will. This thesis is highly debated in the field of ethics (for the latest refutation, see Robichaud 2014). Fitzpatrick opposes the akratic condition. He argues that this condition is too narrow, because it means that people can only be held responsible if an episode of akrasia can be identified at some point in the chain of action. Therefore, he brings in the notions of circumstantial and normative ignorance. Circumstantial ignorance refers to factual ignorance, or lack of knowledge, whereas normative ignorance refers to ignorance of right and wrong. For Fitzpatrick, culpable ignorance implies the exercise of epistemic vices. This will tie in nicely with the ideas developed above on moral responsibility-as-virtue. He writes,

Culpable Ignorance: Ignorance, whether circumstantial or normative is culpable if the agent could reasonably have been expected to take measures that would have corrected or avoided it, given his or her capabilities and the opportunities provided by the social context, but failed to do so either due to akrasia or due to the culpable non-akratic exercise of such vices as over-confidence, arrogance, dismissiveness, laziness, dogmatism, incuriosity, self-indulgence, contempt, and so on. (2008, p. 609)

So culpable ignorance stems from epistemic vices. Therefore, in order to have responsibility-as-virtue for problems involving a lack of knowledge, we can specify responsibility-as-virtue as the responsibility to cultivate epistemic virtues in order to avoid the epistemic vices described above, given that those virtues would be the opposite of those vices. For our case, what is interesting in Fitzpatrick’s definition is the importance of the social context, which makes his theory much more practical for real-life contexts.

Keeping these insights about knowledge and responsibility-as-(epistemic)-virtue in mind, let us now examine a proposal for ascribing forward-looking moral responsibility in the case of genetically modified seeds and consider how these insights can enhance that proposal.

## Current Proposals to Ascribe Moral Responsibility for Contamination of GMOs

In previous work (Robaey 2014), I have suggested ascribing forward-looking moral responsibility to owners of a technology. This proposal was intended to address the hazards of GMOs, given the high level of uncertainty and ignorance with regard to their potential harms. I argue that “the owner of a genetically modified seed has the moral responsibility to do no harm with that seed and there can be several owners of the said seed at the same time that will share moral responsibility for each seed that has the modified character trait and is currently owned” (Robaey 2014, p. 53). In other words, a genetically modified seed has several owners *at the same time*, because each owner will have different sets (or “bundles”) of economic rights over that seed *at the same time*. With ownership rights, however, comes the duty to prevent harm (Honoré 1961). In my work, I make a number of distinctions. First, I use Goodin’s distinction between duties and responsibilities (1986); both notions are prescriptive, but they differ in that duties are a deontological notion, whereas responsibilities are a consequentialist notion. A second useful distinction is the one drawn among risks, uncertainties, and ignorance. Risks are known probabilities for known events, whereas uncertainties are unknown probabilities for known events, and ignorance involves wholly unanticipated factors (Felt et al. 2007). I argue that duties are better suited to dealing with risks, since duties can be formulated as “an agent X should do (or refrain from doing) *α,*” where *α* is a specific action. However, it is very hard to define *α* in situations of uncertainty and ignorance—so an agent X should do something, but that something is not specified and thus requires the agent to have a degree of discretion in order to learn and improvise. Therefore, I argue that it is more useful to speak of responsibilities as a consequentialist notion when dealing with uncertainties and ignorance, i.e. “an agent X ought to see to it that φ” where φ is a desired state of affairs. I suggest transforming Honoré’s ‘duty to prevent harm’ that comes with ownership into a ‘responsibility to do no harm.’ In that sense, ascribing forward-looking moral responsibility both requires and allows owners, i.e. agents who actively decide to use a given technology and have certain rights over it, to monitor and use discretionary powers to bring about a desired state of affairs φ.

The proposal was developed in light of current debates about genetically modified seeds in agriculture. Two main points of contention were identified: (1) about ownership and (2) about risks associated with genetically modified seeds. There was no discussion of moral responsibility for using this technology—thus the proposal to ascribe forward-looking moral responsibility to owners in order to deal with risks. Nevertheless, genetically modified seeds, like other new technologies, pose new challenges. Seeds can self-replicate and spread, so genetically modified seeds can do the same, propagating the modified trait. But saying that owners have forward-looking moral responsibility does not actually solve the problem of contamination.

## The Problem of Contamination

The issue of contamination arises because GMOs are a living technology. The seeds containing the modified gene can replicate and spread; the legal term for this is adventitious presence (AP). Also, the modified genes can be transferred to other plants of the same species or similar species that do not have the modified trait; this is called horizontal gene transfer (HGT). In the realm of GMOs, both these phenomena are commonly known as contamination. I would like to draw an initial distinction here. Recent studies have shown that the risks of HGT between plants are actually very small, and that transfer is more likely to occur with bacteria (Keese 2008). So although HGT has been observed in certain cases (for instance see Beckie et al. 2011), it raises worries only for unmanaged fields (Simard et al. 2002). Since HGT is so rare, we will leave it aside in this paper. Rather, we will focus our analysis on AP, as it has been the source of many lawsuits (cf. Monsanto Canada v. Schmeiser 2001 and OSGATA 2011) and the concern of much legislation, at least in the European Union (EU).

It is interesting to note that AP is commonly referred to as contamination. It is helpful to further investigate the meaning of contamination to understand how it has shaped current regulations of deliberate release and co-existence in the EU (see Regulation (EC) 1829/2003, Regulation (EC) 1830/2003 and special guidelines on co-existence). The focus will be on the EU since it has the most extensive body of regulation for the use of GMOs, which many countries either emulate or criticize for being too strict. It is, however, important to note that the notion of contamination in the food sector is a broad one that generally describes the *unintentional* presence of *undesired* and *potentially dangerous* substances, usually chemicals, bacteria, toxins, or metals, in food or feed. Contamination, which can occur during “production, processing or transport” (EFSA 2015), is therefore a term connected to food safety.

In the case of GMOs, contamination refers to the spread of GMOs to other fields, or to processed foods, which were not supposed to contain them. The European Commission’s website for the Directorate General for Health and Consumers reads: “Conventional products, i.e. those produced without genetic modification, can be contaminated unintentionally by GMOs during harvesting, storage, transport or processing. However, conventional products ‘contaminated’ in this way will not be subject to traceability or labelling requirements if they contain GMO traces below a 0.9 % threshold level, provided that the presence of genetically modified (GM) material is adventitious or technically unavoidable. This is the case where farmers can show the competent authorities that they have taken appropriate measures to avoid the presence of GM material” (DG Health and Consumers).

But where GMOs and food safety are concerned, the use of the term “contamination” is ambiguous. First of all, not all GMOs are the same. The health and/or environmental impacts potentially caused by a flood resistance gene, a pesticide resistance gene, and a gene that codes for vitamin A are unknown, and will most likely not be the same if they do exist. So the spread of GM seeds seems to pose another type of contamination problem, not one necessarily related to food safety. Rather, the spread of GM seeds threatens potential harm in the sense that their presence in other fields, or in other plants, will change the genetic make-up of a given field or plant. This could mean the loss of certain species, and thus loss in biodiversity, or loss in conserving certain species as they were. In the agricultural world, this can result in economic harm to farmers who do not wish to plant GMOs and find their own fields and seeds taken over by unwanted plants. It can lead to the loss of an organic certification, or simply to the loss of the farmer’s or seed breeder’s work in developing a certain crop. This, in turn, can generate problems in food security (rather than food safety) because it can lead to the loss of agricultural biodiversity; large monocultures are more susceptible to biotic shocks, which in the current changing climate could decrease the resilience of agricultural systems (Thrupp 2000, Timmermann and Robaey, forthcoming). Again, it is important to note that all seeds can travel, and spread, and transfer genes, so these problems are not restricted to GMOs. I address the problem via GMOs only because they are the subject of public fear and inquiry.

So there is a problem with GMOs, and it is commonly called contamination. This, as explained above, is a misleading term with regard to the effects of that contamination—but perhaps not when it comes to describing the *unintentional* and *undesired* spread of seeds, as well as the potentially harmful impacts of this spread.

In the European Union, the introduction of GMOs has stirred many fears and controversies (Levidow and Carr 2007), and the notion of co-existence was developed for farmers to be free to choose which kind of seeds they want to use. Plainly put, co-existence suggests that GMO fields and non-GMO fields can exist together, although adventitious presence is said to be technically unavoidable (Levidow and Boschert 2008). Introducing this definition may have created more problems than solutions. Levidow and Boschert argue that the concept of co-existence has in fact given rise to a battle of contradictions between agrarian paradigms (2008). So co-existence did not solve the problem of contamination, i.e. the self-replication and spread of seeds. This boils down to problems of knowledge and control. It is very difficult to predict where GMOs will go and how to trace them (Cardarelli et al. 2005).

There is a growing feeling of injustice regarding how courts deal with disputes over contamination. The case of Monsanto Canada against the Canadian farmer Percy Schmeiser, which was described as one of David versus Goliath, ended in victory for Monsanto. The case of OSGATA, an organic farmer coalition looking to pre-emptively sue Monsanto to prevent them from suing them in case of contamination, saw the same result.

The current system regulating GMOs therefore appears highly inefficient, in that it not only does not deal with potential hazards in a constructive way, but also creates new conflicts and apparent injustices. This puts GMOs, an application of biotechnology with potentially enormous benefits for society, at risk. The problem with contamination is also one of knowledge: who should know where these seeds go, and how should they know?

## Contamination and Ownership

Now that we better understand the nature of the problem of contamination and its implications, let us return to our original problem, namely how the lack of knowledge challenges the ascription of moral responsibility. As mentioned earlier, ascribing forward-looking moral responsibility to owners does not actually solve the problem of contamination. If anything, contamination challenges the very idea of clearly-defined ownership. Indeed, due to the very nature of seeds, which can disperse and self-replicate, the link between ownership and forward-looking moral responsibility is challenged. How do we speak of moral responsibility for technologies that can literally be ‘gone with the wind’? This also problematizes ownership because it would be nonsensical and counterproductive to expect owners to control *all* forces of nature. So do they still bear forward-looking moral responsibility for seeds that might have naturally spread and which they *do not know* about? There is also the case of farmers who might be cultivating those seeds as a result of contamination but also *do not know* of the seeds’ presence in their fields; in a sense, they become de facto owners when they are using the seeds, but does this grant them forward-looking moral responsibility? Another case is one where the genetically modified seeds are in a no-man’s land; then under whose forward-looking moral responsibility do they fall? These problems of ownership also imply problems of ascription of forward-looking moral responsibility for the hazards surrounding GMOs. One can either dismiss the role of ownership in ascribing moral responsibility or strive to further develop this framework. I will do the latter.

If moral responsibility is broadly defined as “an agent X ought to see to it that φ,” where φ is a certain state of affairs in which contamination does not occur as far as reasonably possible, this is rendered difficult in situations of limited knowledge. It is difficult:

1. for original owners to prevent what *they do not and sometimes cannot know*, and
2. for de facto owners to further prevent *what they also do not and perhaps cannot know*.

At this point, it is important to recall that ownership is conceived of as a bundle of rights. In these cases, original owners have different bundles of rights depending on their role and relation to the genetically modified seed. For instance, a farmer may have purchased a seed and have a right to use and derive income from it, but not a right to transfer or a right to possess it (i.e. exclude others from using it). But the company selling the seed may very well have the right to possess it, the right to transfer it, the right to derive income from it, and the right to use it. This is what Honoré calls split ownership, where different agents have different bundles of rights over the same thing. So original owners may have different bundles of rights over the same genetically modified seeds. The de facto owners present an interesting case because they exercise a right to use without knowing that they do. They do not in fact have the right to use, but they unknowingly act as if they did by cultivating a field that might be contaminated. In the GMO case, de facto owners are by default always farmers who did not intend to cultivate GMOs but were cultivating them, like the Canadian farmer Percy Schmeiser.

As explained earlier, with the bundle of rights comes the responsibility to do no harm, which like ownership is also ‘split’ (or shared, or joint). So if the responsibility to do no harm involves preventing contamination, owners have a problem if they don’t know about the contamination.

## Solutions to Contamination: What GMOs can Learn from Nuclear Energy Technologies

GMOs have unique characteristics of self-replication and spread that are not found in other technologies. However, the problems of (1) contamination, (2) shifting ownership (with the de facto owners), and (3) lack of knowledge about effects are not unique to GMOs. Indeed, nuclear energy technologies present similar challenges.

### Contamination

First, nuclear energy technologies have extensively dealt with problems of contamination because of the radioactive wastes they produce. The question of how to deal with nuclear wastes raises several questions relating to safe containment. Before I explain in detail how containment is done in nuclear energy technology, I would like to remind the reader that containment seems to be the most obvious answer to contamination; it is a way of controlling things that spread. This is also why the EU GMO regulations speak of deliberate release and containment.

In the case of nuclear waste, the main concern is how to stop remaining radiations from harming people. Nuclear radiations can spread and can cause harm. There are different kinds of nuclear wastes that require different kinds of storage, but this paper will consider only highly radiotoxic nuclear wastes. Amongst the solutions for dealing with these highly radiotoxic wastes, the most realistic and most commonly used method is deep underground disposal; geological repositories are believed to be the safest locations because they combine engineered and natural barriers. Creating multiple barriers for storage strongly reduces risks of leakage (see Taebi 2012).

While there are similarities to GMOs here, there are also differences. For instance, GMOs do not require that anything similar to nuclear wastes be moved around or stored. Also, in comparison to GMOs, nuclear radiations are generally easier to trace because there will be one point of measurable radioactive emission (unless they leak from the repository). GMOs are usually not easy to find, and tests need to be run on several seeds to establish AP (or contamination). These differences impact traceability, but methods of containment can still be implemented. An interesting lesson from the case of nuclear waste is the idea of multi-layered barriers. What kind of natural and engineered barriers could limit the spread of GMOs? Greenhouses and buffer zones are the ones currently used—especially when required by regulation, as in the European Union—but this is not universal. Maybe the idea of multi-layered barriers can be further implemented at different stages of GMO use in order to reduce the chances of contamination. Also, although there is no waste to be moved, perhaps thinking more strategically about sites of planting will prove helpful.

### Shifting Ownership

Taebi (2012) speaks of inter- and intra-generational issues of justice with regard to the multinational disposal of nuclear wastes. This approach allows one to choose the most apt storage site. Multinational repositories are more just from an inter-generational perspective, because they permit access to the optimal geological repositories, although it is unjust from an intra-generational perspective for one country to take the nuclear wastes of another country. So as one generation makes decisions about the management of these wastes, a future generation will have to deal with these decisions. We can thus observe a shift in ownership of the nuclear wastes.

This is also the problem we see in GMOs. The difference is that the shifting ownership is a direct result of contamination, rather than being due to the longevity of the material in question, as with nuclear wastes. Some of the legal cases described at the beginning of this paper illustrate this shifting ownership. In the case of nuclear wastes, there are institutions and agreements around the idea of multinational waste disposal so that when ownership shifts, new owners have a means of dealing with the wastes. In the case of GMOs, only blame seems to be transferred with ownership; there is no constructive way to deal with them once ownership has shifted.

There are clearly-defined institutions responsible for dealing with nuclear wastes. Due to the different nature of GMOs, there is no such limited range of actors who have clear role responsibilities. Where we would have one nuclear plant and one waste disposal site, we have several farmers using GMOs in different places. This is not necessarily a bad thing. Indeed, Bergen (2015) comments on the need for reversibility in institutional dealings with nuclear wastes. The lack of reversibility might create technological lock-ins where undesirable solutions prevail. In the case of GMOs, it is more the fluidity of technology, of institutions and of actors that prevails. Institutions differ from country to country—and hazards are also perceived differently from country to country, because they are less tangible than the past catastrophes associated with nuclear energy. GMOs have no Three Mile Island, Chernobyl, or Fukushima. This is a good thing. Having overly rigid institutions can create situations that are hard to adapt to, or that make it difficult to change the course of actions if agents find out these actions are wrong. With a more fluid situation, agents can react appropriately without being bound within a system that does not allow for unanticipated change. However, the question remains: if contamination is still such a pressing issue for GMOs, who bears the forward-looking moral responsibility for it, how is it shared, and what does it entail? Practically, this raises the following questions: Where will seeds go? Who will use or own them, intentionally or unintentionally? How will these actors deal with them responsibly?

### Lack of Knowledge About the Effects

The third point of comparison is with regard to the effects of GMOs and radiation from nuclear wastes. There are many unknowns as to what will happen, and where and when it will happen, with nuclear wastes thousands of years down the line. Shrader-Frechette (1993) argues that the knowledge we have consists only of predictions stretching over 10,000 years, and that it is not reliable. In the same way, there are many unknowns as to the long-term effects of certain GMOs on the environment and on human health.

Pondering the case of nuclear energy brings us to a much-discussed notion: the social experiment. This concept has had many meanings in the past, both normative and policy-related. In the case of new technologies, it has had pejorative connotations. After Chernobyl, Krohn and Weingart (1987) wrote about the nuclear social experiment in a negative way. Indeed, society was experimenting, which also meant making mistakes at a scale where humans could be severely harmed. Similar analogies have been made in the realm of biotechnology, referring to GMOs as Frankenfoods (Van den Belt 2009) or as a “gigantic experiment” (BBC 2008). Beck (1992) describes new risks as unintentional, unseen, and compulsive; such risks create new societal problems. In the recent literature, non-pejorative descriptions of GMOs as experimental also appear, at least in terms of regulatory experiments (cf. Levidow and Carr 2007; Millo and Lezaun 2006). In the fields of ethics of risk and engineering ethics, Van de Poel (2011a) takes a stance on the nuclear catastrophe of Fukushima by arguing that we *should* experiment. Here, the notion of the social experiment is a normative one. Indeed, we should experiment in society because then we have to follow certain ethical conditions for responsible experimentation (Van de Poel 2011a). By experimenting, agents have to fulfill both higher and instrumental values. For instance, pursuing instrumental values such as learning and intervening will allow us to fulfill higher values such as justice, autonomy, benevolence, and non-maleficence to all members of society (Robaey and Simons 2015).

What is unusual about Van de Poel’s use of the term “social experiment” is that it emphasizes the unknowns that remain when we use new technologies. Unlike classical experiments, social experiments are not controlled and limited by one experimenter. Many agents are experimenting, and many may not be aware that they are experimenting. Being part of a social experiment gives rise to special responsibilities to fulfill certain conditions or values. Van de Poel’s proposal draws on literature in bioethics and environmental management. It does not define responsibility per se, but rather defines the aims of responsible introduction of new technologies into society: namely, minimizing negative and unwanted side effects to make the best of technologies that can greatly improve our lives.

In the next section, I ask how this notion of a normative social experiment relates to using ownership as a means of ascribing moral responsibility, and I explore the idea that owners must develop epistemic virtues in order to be responsible.

## Owners as Social Experimenters: Some Practical Recommendations

Owners are social experimenters because they are the ones who actively decide to develop and/or use technologies, in this case GMOs. Who are the owners, and what should an owner do as a social experimenter?

First of all, not all owners will have the same interaction with the seeds, so they may have different means of pursuing the instrumental value of intervening. This also applies to learning, as well as to the other higher values of responsible experimentation. Indeed, those values will translate to different norms and requirements for different actors (Van de Poel 2013). It is this complementarity and concerted action that will in the aggregate fulfill the goal of shared forward-looking moral responsibility for achieving a desired state of affairs φ, where contamination does not occur (or its chances of happening are greatly reduced).

The fluidity of institutions mentioned in the previous section might appear problematic; yet it can, under the right conditions, prove to be a very strong asset. If they are to bear forward-looking moral responsibility, owners should have self-supervisory and discretionary power, following Goodin’s consequentialist definition of responsibility. Therefore, they are responsible for learning about the technology they develop and use in order to be able to react to unexpected events. As explained at the beginning of this paper, we choose to speak of responsibilities because duties are too limiting in terms of the instrumental values of learning and intervening (to use the language of the social experiment).

Also, if we formulate forward-looking moral responsibility as “an agent X ought to see to it that φ,” φ takes on a new meaning in the social experiment. Under uncertainty, φ seems more likely to be achieved if the experiment is carried out responsibly. In addition, lacking knowledge about the presence of seeds does not absolve owners of their responsibilities but instead further defines these responsibilities. Since owners can carry out the social experiment responsibly regardless of the outcome and regardless of their lack of knowledge, the problem of contamination no longer challenges the *allocation* of responsibilities according to ownership. It is then a collective, or joint, imperative to act responsibly to limit or avoid contamination.

To refine the notion of the social experiment, Van de Poel (2011a) suggests initial conditions for responsible experimentation (see Table 1). As mentioned, these conditions can be linked back to a set of instrumental and higher values. In a way, these conditions refer to a range of actions that actors, or in this case owners, may take in order to fulfill their forward-looking moral responsibility. In this paper, we are concerned specifically with the problem of contamination and with the role of owners—in other words, with how forward-looking moral responsibility works for a technology that spreads in an unexpected way. Therefore, we will focus on asking what the conditions of *monitoring, scaling*-*up and flexible set up,* and *containment of hazards as far as reasonably possible* can mean as a range of actions for the different types of owners involved. There are several other conditions presented by Van de Poel that will not be elaborated on in this paper due to space constraints. In the following paragraphs, I explore what these conditions imply for owners’ range of actions. And I will later link these findings to owners’ epistemic virtues. It is important to distinguish among the different types of owners in order to establish what they can do, or what their capacities are.

**Table 1:** Possible conditions for socially responsible experimentation (Van de Poel 2011a)—emphasis added to the conditions under study

1. Absence of other reasonable means for gaining knowledge about hazards
**2. Monitoring**
3. Possibility to stop the experiment
**4. Consciously scaling up**
**5. Flexible set-up**
6. Avoid experiments that undermine resilience of receiving ‘system’
**7. Containment of hazards as far as reasonably possible**
8. Reasonable to expect social benefits from the experiment
9. Experimental subjects are informed
10. Approved by democratically legitimized bodies
11. Experimental subjects can influence the set-up, carrying out and stopping of the experiment
12. Vulnerable experimental subjects are either not subject to the experiment or are additionally protected
13. A fair distribution of potential hazards and benefits

Owners are not only the ones who decide to use a given technology, but also the ones who have certain rights over it. Moreover, it is important to think about which of these conditions apply to owners, as some (such as approval by democratically legitimized bodies) are clearly meant for other social actors. Indeed, owners are not the only experimenters. Citizens, governments, agencies, and NGOs all participate in the social experiment; they are affected by and involved with the technology in society.

If we consider scientists or bio-engineers to be the primary developers of GMOs, for them the condition of monitoring could mean providing guidance about what to monitor. Also, they could integrate markers that would make finding GMOs easier. In the past, fluorescence genes have been suggested, but other ideas could be developed as well. For the conditions of the set-up and the scaling-up, developers are not in the field, so their range of actions is limited. Finally, with regard to containment of hazards, developers can think in terms of design requirements—this time not for the seeds themselves but for their management in the field.

In many ways, farmers have the same responsibilities as scientists. For instance, they must provide local knowledge for identifying what to monitor and must consider design requirements for using field management to contain hazards, which goes hand-in-hand with the idea of the flexible set-up and scaling-up. Farmers have first-hand knowledge and experience of their fields, and they should be given discretionary power to set these up in the way they feel is safest. By learning from factors such as wind patterns and migration habits of local species, they can integrate new knowledge into the management of their fields. Their position at the front line, so to speak, gives them increased burdens. However, there should be support structures to prevent them from becoming over-burdened as they seek to carry out their forward-looking moral responsibility.

If a farmer becomes an unintentional de facto user by harvesting seeds without knowing that they are GMOs, it is a bit trickier to define her range of actions. However, if these farmers are aware of the proximity of other seeds and aware that they could become de facto owners, then they can support the GMO farmers in providing information and constructing barriers. Coordination among different actors will help to resolve the issue of contamination. The burdens of potential de facto owners should not be greater than the ones of original owners. However, in the current system, these de facto owners are unfairly over-burdened with backward-looking responsibility. Unfortunately, their cooperation is necessary to head off contamination and to react to it quickly if it does occur.

This is where companies, universities, and research institutes who are promoting and benefitting from the applications of GMOs have a special role. As institutions, they are not necessarily “on the ground” as scientists and farmers can be, so their role is a supportive one that involves facilitating and aiding proper implementation. Currently, companies like Monsanto spend a lot of resources searching for their seeds in others’ fields, as several lawsuits have demonstrated. They could spend the same resources to help create multi-layered barriers, as we have learned from the case of nuclear wastes.

I have mentioned design requirements for GMOs. One goal is to address contamination, but this should not hinder achieving values of benevolence; in the case of seeds, benevolence is about creating agricultural systems that are sustainable and can feed the world. So design requirements should not produce more problems, as with the concept of the Terminator gene (Van den Belt 2009). Companies, universities and research institutes can play a role in providing a good (i.e. benevolent) direction for the people who work for them in developing GM seeds.

Retailers and seed distributors have a different kind of role in that they help to spread seeds. However, like the institutions mentioned above, they are not directly in the field. Their role can instead be supportive. They can make sure that purchasers of GM seeds, like farmers and consumers, are well aware of how to deal with them in terms of limiting contamination. This might, for instance, involve training and appropriate labelling.

These practical recommendations, derived from the concept of the social experiment, are strengthened by the notion of forward-looking moral responsibility as epistemic virtue. The next section shows exactly how.

## Taking Responsibility Under Uncertainty

This paper began by posing the following question: if we cannot know of contamination, how can we ascribe forward- and backward-looking responsibility for it to owners? Now that we have seen in more practical detail what an owner can do as a responsible experimenter, I would like to return to the notion of responsibility as virtue. Indeed, the three conditions for morally responsible experimentation that we translated into different ranges of action for each actor all relate to the development of epistemic virtues, regardless of the type of owner involved. Be it through new design requirements to enhance traceability, or be it through new ways to learn about dispersion in order to better anticipate and limit it, these actions are the result of developing epistemic virtues.

Earlier, we listed what FitzPatrick calls epistemic vices, but we did not go into further detail once we established that virtues are the opposite of vices. Early discussions of epistemic virtues were linked to belief formation and finding the truth. While these are relevant questions, they are outside the scope of this paper, which focuses on practical applications. For now, it is sufficient to mention what Montmarquet (1987) identifies as possible epistemic virtues: impartiality, intellectual courage, and community. He also warns that epistemic virtues should be regulated, because otherwise they might turn into dogma; hence the need for community. In other words, an agent who develops and uses a new technology is responsible for finding out more about it in a sound way and for sharing that knowledge. To use the vocabulary of the social experiment, epistemic virtues allow an owner to fulfill the instrumental values of learning and intervening—and thus possibly other higher values.

Yet this might seem like a lot to demand of certain agents. In the previous section, we distinguished among various roles. For instance, one can hope that scientists who have the role of developers will possess epistemic virtue, given that they work in the field of research. In this case, the key is to expand their scope of inquiry, which we suggested via the notion of social experiments. Similar considerations apply to actors who can be considered sponsors or spreaders; their curiosity should not stop at the development and deployment of the product, but should also apply to the use of the product, to see whether it actually helps to bring about their goals. As for farmers, it seems that they are on the front line of the experiment and can observe more. They might therefore bear greater burdens when it comes to developing epistemic virtues. Epistemic demands might be too much for certain agents to carry out, which would create a responsibility gap.

This is where Fitzpatrick’s definition is especially important. He writes that ignorance is culpable if an agent fails to do what he or she could “given his or her capabilities and the opportunities provided by the social context” (2008, p. 609). Similarly, in the context of backward-looking moral responsibility, David Miller proposes the capacity principle: “remedial responsibilities ought to be assigned according to the capacity of each agent to discharge them. […] If we want bad situations put right, we should give the responsibility to those who are best placed to do the remedying” (2001, pp. 460–461). These distributive ideas are very clear from the standpoint of backward-looking moral responsibility. Can we translate them to a forward-looking allocation of responsibility? Of course being culpable for a wrong, or being responsible for remedying a wrong, is not the same as having forward-looking moral responsibility for something that didn’t happen. However, the contexts and circumstances surrounding the use of GM seeds are not unknown. If each actor’s range of actions and responsibilities as epistemic virtues are at the level of that actor’s capacity to carry them out—in a manner that their epistemic virtues will help them define—then they are able to take responsibility for the technology they are developing or using. How owners’ responsibilities relate to each other is beyond the scope of this paper, but it is certainly a topic for future investigation.

## Conclusion

GMOs promise improved agricultural yields in both quantity and quality. However, their use has proven to be highly contested and problematic. In this paper, I examined the proposal that we should allocate forward-looking moral responsibility to owners of GMOs, as well as the limits of this proposal in the case of contamination. Contamination highlights the problems stemming from limited or non-extant knowledge about the spread of GMOs, and therefore challenges the ascription of moral responsibility. I established that incomplete knowledge does not remove forward-looking moral responsibility from owners. GMOs are a technology that can self-replicate and easily spread because of the nature of seeds. I focused on two problematic cases: (1) the responsibility of original owners with regard to potential contamination, and (2) the responsibility of de facto owners. Looking at nuclear wastes led to two observations: (1) GMOs could benefit from multi-layered barriers, and (2) the normative notion of the social experiment provides owners with a range of actions. Through this reasoning, a range of action was defined for different types of owners. This provides a solution to case (1), as the responsibility of owners to avoid contamination is further defined by shared values relating to responsible experimentation. Therefore, owners are responsible for developing epistemic virtues and defining a range of actions to deal with potential problems, such as contamination. Lack of knowledge does not pose a problem for the ascription of moral responsibility, because owners are experimenters who should take responsibility.

This paper focused on forward-looking moral responsibility; this does not, however, mean that backward-looking responsibility is not important. Indeed, mechanisms of backward-looking moral responsibility can influence agents’ forward-looking responsibility (cf. Van de Poel 2011b). In the case of GM seeds, several lawsuits have transferred backward-looking responsibility to unintended owners. But if conditions of forward-looking moral responsibility had been fulfilled by the original owners, we would be left with cases that are straightforwardly morally wrong, such as theft of seeds, rather than unintentional cases. This framework does not change the rights of owners, but instead suggests forward-looking responsibilities that provide a fair and efficient paradigm where stealing (rather than unintentional use) is punished. Owners developing epistemic virtues and defining their range of actions will allow for more responsible use of GMOs, despite incomplete knowledge. Seeds will then not simply be gone with the wind, because owners will take responsibility and do everything they can to avoid contamination.
